# ICNP^®^ terminological subset for preventing falls in the elderly in primary care

**DOI:** 10.1590/1980-220X-REEUSP-2022-0483en

**Published:** 2024-02-02

**Authors:** Paulo Henrique Fernandes dos Santos, Marina Morato Stival, Luciano Ramos de Lima, Cris Renata Grou Volpe, Silvana Schwerz Funghetto

**Affiliations:** 1 Universidade de Brasília, Departamento de Enfermagem, Brasília, DF, Brazil.; 2 Universidade de Brasília, Faculdade de Ceilândia, Brasília, DF, Brazil.

**Keywords:** Nursing, Standardized Nursing Terminology, Aged, Accidental Falls, Primary Health Care, Enfermería, Terminología Normalizada de Enfermería, Anciano, Accidentes por Caídas, Atención Primaria de Salud, Enfermagem, Terminologia Padronizada em Enfermagem, Idoso, Acidentes por Quedas, Atenção Primária à Saúde

## Abstract

**Objective:**

Build and validate a terminological subset of ICNP^®^ for the prevention of falls in the elderly in the context of primary health care, in light of the Self-Care Deficit Theory.

**Method:**

Methodological study developed in accordance with ICN recommendations and the Brazilian method for constructing terminological subsets, in two stages: 1) construction of ICNP^®^ statements of nursing diagnoses, outcomes, and interventions; 2) content validation of statements by specialist nurses.

**Results:**

A total of 182 diagnoses/outcomes and 321 nursing interventions were constructed, which were subjected to content validation by 28 experts, being validated with a Content Validity Index ≥ 0.80. After validation, the statements were organized according to self-care requirements and the majority of diagnoses/outcomes (51.6%) and interventions (52.7%) were classified under health deviation requirements.

**Conclusion:**

It was possible to construct and validate a terminological subset of ICNP^®^ with a predominance of statements related to health deviation requirements, standing out for being the first terminological subset for the prevention of falls in the elderly in the context of primary care.

## RESUMO

**Objetivo:**

Construir e validar um subconjunto terminológico da CIPE^®^ para a prevenção de quedas em idosos no contexto da atenção primária à saúde, à luz da Teoria do Déficit do Autocuidado.

**Método:**

Estudo metodológico desenvolvido conforme recomendações do ICN e do método brasileiro para construção de subconjuntos terminológicos, em duas etapas: 1) construção de enunciados de diagnósticos, resultados e intervenções de enfermagem da CIPE^®^; 2) validação de conteúdo dos enunciados por enfermeiros especialistas.

**Resultados:**

Foram construídos 182 diagnósticos/resultados e 321 intervenções de enfermagem, os quais foram submetidos a validação de conteúdo por 28 especialistas, sendo validados com Índice de Validade de Conteúdo ≥ 0,80. Após a validação, os enunciados foram organizados conforme os requisitos de autocuidado e a maioria dos diagnósticos/resultados (51,6%) e das intervenções (52,7%) foi classificada nos requisitos de desvio de saúde.

**Conclusão:**

Foi possível construir e validar um subconjunto terminológico da CIPE^®^ com predomínio de enunciados relacionados aos requisitos de desvio de saúde, destacando-se por ser o primeiro subconjunto terminológico para a prevenção de quedas em idosos no contexto da atenção primária.

## RESUMEN

**Objetivo:**

Construir y validar un subconjunto terminológico de la CIPE^®^ para la prevención de caídas en ancianos en el contexto de la atención primaria de salud, a la luz de la Teoría del Déficit de Autocuidado.

**Método:**

Estudio metodológico desarrollado de acuerdo con las recomendaciones del CIE y el método brasileño de construcción de subconjuntos terminológicos, en dos etapas: 1) construcción de declaraciones de diagnóstico, resultados e intervenciones de enfermería de la CIPE^®^; 2) validación de contenido de declaraciones de enfermeros especialistas.

**Resultados:**

Se construyeron 182 diagnósticos/resultados y 321 intervenciones de enfermería, los cuales fueron sometidos a validación de contenido por 28 expertos, siendo validados con un Índice de Validez de Contenido ≥ 0,80. Después de la validación, los enunciados fueron organizados según los requisitos de autocuidado y la mayoría de los diagnósticos/resultados (51,6%) y las intervenciones (52,7%) fueron clasificados bajo requisitos de desviación de salud.

**Conclusión:**

Fue posible construir y validar un subconjunto terminológico de la CIPE^®^ con predominio de enunciados relacionados a requerimientos de desviación de salud, destacándose por ser el primer subconjunto terminológico para la prevención de caídas en el anciano en el contexto de la atención primaria.

## INTRODUCTION

Falls, defined as events in which the individual inadvertently falls to the ground or to another level lower than the initial position^(1)^, represent one of the greatest threats to the well-being of the elderly, resulting in economic, social and psychological consequences. They are responsible for high hospitalization rates, negative repercussions on functional capacity and independence, social isolation, and death^(2)^.

Despite affecting the entire population, falls affect the elderly more significantly. The frequency of these events increases with increasing age and the level of frailty of individuals; data indicate that around 28% to 35% of elderly people over 65 years of age experience at least one fall each year, a proportion that rises from 32% to 42% for elderly people over 70 years of age, and up to 50% for those aged 80 and over^(3)^.

The occurrence of falls has multifactorial causes and risk factors encompass biological, behavioral, environmental, and socioeconomic dimensions^(3)^, which demands a comprehensive approach with an emphasis on prevention actions. Therefore, the involvement of nurses who work in primary health care (PHC) in approaching elderly people to prevent falls is essential, considering that care allows the development of educational skills and contributes to the construction of knowledge and encouragement of the adoption of healthy habits at the individual and collective level^(4)^.

The nurses’ work is operationalized through the Nursing Process (NP), a methodological instrument that allows critical analysis, guides clinical judgment, decision-making regarding the client’s health, the effective performance of the nursing team, in addition to promoting autonomy, independence, and specificity of the profession. The NP is structured in the interrelated stages of investigation, nursing diagnosis, planning, implementation and evaluation, which are implemented based on a theoretical framework, generally a nursing theory^(5)^.

One example is the Self-Care Deficit Theory (SCDT), proposed by Dorothea Elizabeth Orem in 1971. SCDT is expressed in three interrelated theories – self-care theory, self-care deficit theory, and nursing systems theory, and is based on the premise that all individuals have the potential to take care of themselves. According to Orem, when the individual presents an imbalance between the ability to exercise self-care and the therapeutic demands of self-care, the nurse can assume these activities partially or completely, in addition to offering educational support^(6)^.

In addition to being based on theoretical references, another aspect that permeates the NP is the standardization of language through nursing classification systems, for example, the International Classification for Nursing Practice (ICNP^®^) designed by *International Council of Nurses* (ICN). ICNP^®^ includes nursing diagnoses (ND), nursing outcomes (NO), and nursing interventions (NI), in addition to allowing the creation of new statements, according to the users’ specificities. From this perspective, the ICN guides the construction of terminological subsets aimed at certain clientele, health priorities or nursing phenomena, based on theoretical references^(7)^.

With regard to the elderly population, terminological subsets of ICNP are identified in the literature^®^ for older patients^(8)^, institutionalized older patients^(9)^ and for elderly women with vulnerability related to HIV/AIDS^(10)^. Regarding falls, a subset was identified aimed at preventing falls for the general public^(11)^. However, there is a gap in the national and international scenario in relation to terminological subsets and the development of other instruments that contribute to nurses’ actions in preventing falls in the elderly in the context of PHC.

Therefore, due to the relevance of the phenomenon of falls in the context of aging, and the potential role of nurses in preventing these events in primary care, it is considered essential to propose a standardized language that facilitates and improves the work of these professionals. The instrument has the potential to facilitate access to ND/NO/NI related to the prevention of falls in older people; to generate data to support clinical practice, decision making, research and professional training, in light of a nursing theory; to enable standardized documentation of care; to increase the visibility of nursing in services and information systems; in addition to contributing to the expansion of the use of ICNP^®^.

In the construction of this study, SCDT was adopted as a theoretical reference, a choice justified by the diversity of the profile of users served in PHC, considering older people with different abilities to perform self-care; the need to promote the development of skills to carry out self-care actions in older people and their families with the aim of preventing episodes of falls; the role of nurses at this level of health care, with an emphasis on health promotion, protection and prevention of injuries. The fact that there are no ICNP^®^ terminological subsets in the literature for the prevention of falls in this specific population, from the perspective of self-care, reveals the originality and innovation of the work.

In view of the above, the following guiding question was elaborated: which statements of ND, NO, and NI can compose a terminological subset of the ICNP^®^ for the prevention of falls in the elderly in the context of PHC, based on SCDT? Therefore, the study aims to build and validate a terminological subset of the ICNP^®^ for the prevention of falls in the elderly in the context of PHC, in light of SCDT.

## METHOD

### Design of Study

Methodological study, conducted from August 2021 to May 2022. Two stages were carried out, in accordance with recommendations from the ICN and the Brazilian method for developing ICNP terminological subsets^®(12)^: 1) construction of CIPE^®^ ND, NO, and NI statements for preventing falls in elderly people in PHC, based on specialized terminology previously constructed^(13)^; 2) content validation of statements by specialist nurses.

### Population and Selection Criteria

For content validation, specialist nurses were selected according to the criteria proposed by Fehring^(14)^, with adaptations relevant to the study theme: master’s degree in nursing (4 points); master’s degree in nursing, with thesis in the areas of interest (1 point); research on classification systems in nursing and/or gerontology (2 points); published article on classification systems in nursing and/or gerontology (2 points); doctorate in nursing, with a dissertation in the areas of interest (2 points); clinical experience, of at least one year, in healthcare for the elderly in PHC or other levels of care (2 points); specialization in areas of interest (2 points). Experts who achieved at least 5 points were selected.

The search for experts took place on the Lattes Platform of the National Council for Scientific and Technological Development (CNPq); in the CNPq Research Group Directory; on websites of national undergraduate and postgraduate nursing courses; in addition to indication by other research participants (“snowball” technique). The searches resulted in a list of 238 possible participants, who were recruited by sending an invitation letter via email in January 2022.

### Sample Definition

To define the sample, the formula n = Z^2^ 1-α/2. p.(1–p) /e2 was used, where “Z^2^ 1-α/2” = confidence level; “p” = expected proportion of experts; and “e” = acceptable proportion difference in relation to what would be expected^(15)^. A confidence level of 95% (Z^2^ 1–α/2 = 1.96) was considered, an expected proportion of 85% of experts and a sampling error of 15%, resulting in an ideal sample of 22 experts. Considering the difficulties in obtaining responses from experts, it was decided that the invitation letter would be sent to an expanded list of possible participants, with the expectation of reaching at least 22 participants.

### Data Collection

Data collection took place from August 2021 to March 2022. In the first stage, the ND/NO/NI statements were constructed, based on the specialized terminology elaborated by the authors previously^(13)^, the terms contained in the ICNP^®^ Seven Axis Model version 2019, ISO 18.104 standards^(16)^, SCDT and scientific literature.

Initially, the ND/NO related to the prevention of falls in elderly people in PHC were constructed, necessarily including a term from the “Focus” axis and a term from the “Judgment” axis; additional terms from other ICNP^®^ axes were added as necessary. The ND/NO were organized in alphabetical order in a spreadsheet *Excel for Windows* ^®^ and subjected to cross-mapping with the ND/NO concepts proposed by ICNP^®^. The mapping was carried out in *Access for Windows* ^®^ and generated a list of constant and non-constant ND/NO in the classification.

Non-constant ND/NO underwent analysis of the grade of equivalence according to the criteria established in ISO/TR 12.300:2016^(17)^: grade 1 – lexical and conceptual meaning equivalence; grade 2 – equivalence of meaning, but with synonymy; grade 3 – source concept is broader and has less specific meaning than the target concept/term; grade 4 – source concept is more restricted and has more specific meaning than the target concept/term; grade 5 – no mapping is possible.

Next, operational definitions for ND/NO were developed with the aim of communicating their meanings and explaining how to measure them. For ND/NO listed in ICNP^®^, the operational definitions contained in the terminology were transcribed and, when relevant, textual changes were made consistent with the theme of the terminological subset, for example, to specify the elderly clientele. In this process, the descriptions of the ND/NO as well as the descriptions of the terms of the Focus axis were used as a basis. For ND/NO not included in ICNP^®^, operational definitions were developed considering the descriptions of the ICNP^®^ primitive and pre-arranged terms version 2019, NANDA-I, dictionaries of the Portuguese language, and technical terms in the area of health and nursing, books on semiology and semiotechnique, books on nursing theories, in addition to scientific articles.

It should be noted that the ND/NO were subjected to a prior classification according to the SCDT self-care requirements, that is, universal, developmental, and health deviation requirements^(6)^.

Next, NIs aimed at ND/NO were constructed, including a term from the “Action” axis and a target term (terms from any of the axes, except “Judgment”). These were organized in alphabetical order in a spreadsheet from *Excel for Windows* ^®^, standardized in terms of spelling, gender inflections, number, degree, in addition to being standardized with the ICNP^®^ NI and subjected to cross-mapping with the NI concepts proposed by ICNP^®^, through the *Access for Windows* ^®^, resulting in a list of constant and non-constant NI. The non-constant NI were subjected to analysis of the degree of equivalence according to the criteria of ISO/TR 12.300:2016^(17)^ and organized according to the classification of their corresponding ND/NO.

The second stage consisted of validating the content of the experts’ statements. Initially, data collection instruments were created in electronic format, using Google Forms. The first consisted of the identification of participants and the Free and Informed Consent Form (FICF). The second consisted of: characterization of the experts; guidelines for evaluating ND/NO/NI; lists of ND/NO, with their respective operational definitions, organized according to self-care requirements and the respective items for evaluating content and suggestions; lists with the NI, assigned to the ND/NO set of each self-care requirement, with items for evaluating the content and suggestions; field to indicate the contact details of other nurses to receive an invitation to participate in the research.

The ND/NO/NI were assessed using a Likert-like scale with a score from 1 to 4, with the definitions: 1. non-significant or non-representative item; 2. item requires major revision to be significant/representative; 3. item requires minor revision to be significant/representative; 4. significant and representative item. For items rated “2” or “3”, there was a field for sending suggestions. In relation to ND/NO, experts were asked whether the statements were adequately classified in the self-care requirements; if the answer was negative, they could objectively indicate which requirement the statement should be reallocated to.

During the first contact with the experts, the link to sign the FICF was made available. After consent, the link to the data collection instrument was sent, indicating a period of 30 days to complete participation in the study. Following content validation, the ND/NO/NI statements were organized to compose the terminological subset, considering the self-care requirements suggested by SCDT: universal requirements, development requirements, and health deviation requirements.

### Data Analysis and Treatment

At the end of data collection, in March 2022, the results were compiled in a spreadsheet from the *Excel for Windows* ^®^, aiming to consolidate the database. The characterization data were analyzed with descriptive statistics (frequency and percentages); and to measure experts’ agreement regarding the validity of ND/NO/NI, the Content Validity Index (CVI) was adopted. The CVI score was calculated considering the items evaluated with a score of 3 or 4, divided by the total sum of responses to the items. ND/NO/NI with CVI ≥ 0.8 were validated^(18)^. In addition to statistical analysis, suggestions for reviewing statements, operational definitions, and classification of self-care requirements sent by experts were analyzed, with necessary/relevant adjustments being made.

### Ethical Aspects

The study was approved by the Research Ethics Committee of the School of Ceilândia – University of Brasília, opinion 3.693.419, in 2019, in compliance with Resolution 466/12 of the National Health Council and the guidelines for procedures in research with stages in a virtual environment published by the National Research Ethics Commission. Data collection was carried out after participants signed the FICF.

## RESULTS

In the first stage, 182 ND/NO statements related to falls prevention were constructed. The list of ND/NO prepared was cross-mapped with the ND/NO concepts proposed by the ICNP^®^, the result of which showed 104 constant statements and 78 non-constant statements. The non-constant ND/NO were subjected to analysis of the degree of equivalence, in which 25.6% (n = 20) were classified as grade 2, 1.3% (n = 1) as grade 3, 11.5% (n = 9) as grade 4, and 61.5% (n = 48) as grade 5. It should be noted that the statements contained in the ICNP^®^ (n = 104) were assigned grade 1 of equivalence. The ND/NOs classified as grade 2 (n = 20) were replaced by the corresponding ICNP terms^®^ and added to the previous list of constant statements, resulting in 124 constant ND/NO. In their turn, ND/NO classified as grades 3, 4 and 5 were considered non-constant (n = 58).

After the equivalence analysis, operational definitions were developed for the constant ND/NO that did not have definitions in ICNP^®^ and for ND/NO not included in the terminology. Once the elaboration of the operational definitions was completed, a prior classification of the ND/NO was carried out according to the self-care requirements, with 69.6% (n = 127) classified under the health deviation requirements, 15.5% (n = 28) in universal requirements, 14.9% (n = 27) in development requirements.

Based on the ND/NO, 321 NI were constructed related to the prevention of falls in older people in PHC. The list of interventions constructed was organized in alphabetical order and subjected to the cross-mapping process with the ICNP^®^ NI concepts. The result of the mapping was 213 constant statements and 108 non-constant statements. The non-constant statements were subjected to analysis of equivalence, in which 14.8% (n = 16) were classified as grade 2, 1.9% (n = 1) as grade 3 (n = 2), 6.5% (n = 2) as grade 4 (n = 7), and 76.9% as grade 5 (n = 83). To the NI contained in the ICNP^®^ (n = 213) grade 1 of equivalence was assigned. The NI classified as grade 2 (n = 16) were replaced by the corresponding ICNP concepts^®^ and added to the previous list of constant statements, resulting in 229 constant NI. In their turn, NI classified as grades 3, 4 and 5 were considered non-constant (n = 92).

Once the analysis of the grade of equivalence was completed, a prior assignment of the NI to the ND/NO was carried out in accordance with the self-care requirements. Of the total NI (n = 321), 63.8% (n = 205) were assigned to ND/NO classified under health diversion requirements, 23.4% (n = 75) to ND/NO classified under universal requirements and 12.8% (n = 41) to ND/NO classified in the development requirements.

In the second stage, the invitation letter and the FICF were sent to 238 nurses, of which 53 signed the FICF and 28 completed participation. Therefore, validation was carried out by a sample of 28 specialist nurses, of which 85.7% were female, with a predominant age range of 26 to 35 years (46.4%), with training time of more than 10 years (71.4%). Regarding work, there was a predominance of nurses who work in teaching (96.4%), research (89.3%) and extension (64.3%). Furthermore, 75.0% of the participants declared that they had a master’s degree in nursing, 75.0% had already published articles on classification systems in nursing and/or gerontology, 71.4% carried out or had already carried out research on these topics, 67.9% have clinical experience in older people health care in PHC and 53.6% have a doctorate in nursing and have researched topics related to the present study. Nurses from the five regions of Brazil participated, especially from the Northeast (35.7%), Center-West (25.0%), and South (17.9%).

The content validation results showed the validation of all ND/NO/NI, that is, 182 ND/NO and 321 NI. After reviewing the classification of statements regarding self-care requirements, according to experts’ suggestions, of the total ND/NO (n = 182), 51.6% (n = 94) were classified under health deviation requirements, 33.5% (n = 61) in universal requirements, and 14.9% (n = 27) in development requirements. As for NI (n = 321), 52.7% (n = 169) were assigned to the ND/NO of health diversion requirements, 35.2% (n = 113) to universal requirements and 12.1% (n =39) to development requirements.

Next, the ND/NO/NI were organized according to self-care requirements and each ND was assigned one (or more) NO and a list of NI. In this sense, the subset consisted of 92 ND, 90 NO, and 321 NI; the statements contained in ICNP^®^ were presented with their respective codes (Table 1).

**Table 1:** Examples of ICNP nursing diagnoses, outcomes, and interventions^^®^^ for preventing falls in the elderly in the context of primary health care organized according to self-care requirements and the respective Content Validity Indexes (CVI) – Brasília, DF, Brazil, 2023.

NURSING DIAGNOSES/OUTCOMES, AND INTERVENTIONS	CVI
**UNIVERSAL SELF-CARE REQUIREMENTS**	
**Nursing Diagnosis/Outcome** Risk for Fall (10015122) Risk for Fall, Absent **Nursing Intervention** Demonstrating Falls Prevention (10040248) Recommend Environment with Adequate Lighting Recommend Anti-Slip Mat Remove Obstacles (Objects, Furniture) from the Floor Assessing Risk for Falls (10023520)	1.00 1.00 1.00 1.00 1.00 1.00 0.93
**Nursing Diagnosis/Outcome** Impaired Exercise Behavior (10022043) Physical Exercise Behavior, Effective **Nursing Intervention** Assess Adherence to the Physical Exercise Regimen Guidance on Sedentary Lifestyle Teaching about Exercise (10040125) Manage Exercise Regime (10023890) Get Physical Fitness Data	0.93 0.93 1.00 1.00 1.00 0.93 0.93
**Nursing Diagnosis/Outcome** Self-Care Deficit (10023410) Able to Perform Self Care (10025714) **Nursing Intervention** Teaching about Self Care (10045014) Promoting Self-Care (10026347) Assisting with Self-Care (10035763) Establish Self-Care Routine Identify Barriers to Self-Care in the Nursing Consultation	0.93 0.93 1.00 1.00 0.96 0.96 0.96
**Nursing Diagnosis/Outcome** Lack of Knowledge of Fall Prevention (10040230) Knowledge of Fall Prevention (10040276) **Nursing Intervention** Guide the Nursing Team on Fall Prevention Teaching Family about Fall Prevention (10040269) Promote Educational Action on Fall Prevention Assessing Readiness to Learn (10002781) Providing Instructional Material (10024493)	0.93 0.93 1.00 1.00 1.00 0.96 0.96
**Nursing Diagnosis/Outcome** Environmental Safety Problem (10029856) Effective Environmental Safety (10030233) **Nursing Intervention** Identify Stairs or Steps in the Home Implementing Safety Regime (10036565) Assessing Environment (10026064) Teaching About Safety Measures (10024687) Evaluating Home Before Home Care (10041038)	0.93 0.93 1.00 1.00 0.96 0.96 0.89
**SELF-CARE REQUIREMENTS RELATED TO DEVELOPMENT**	
**Nursing Diagnosis/Outcome** Impaired Psychomotor Activity (10025087) Psychomotor Activity, Improved **Nursing Intervention** Facilitating Activity of Daily Living (10051125) Refer to Physiotherapy Monitoring Fall Risk (10037442)	0.96 0.96 0.96 0.93 0.93
**Nursing Diagnosis/Outcome** Impaired Balance (10047170) Improved Balance (10047348) **Nursing Intervention** Obtain Data on Proprioceptive Deficit Assessing Balance (10037457) Obtain Data on Postural Instability Advise on the Risk of Rapid Body Movements Promoting Adherence to Exercise Regime (10041628)	0.96 0.96 0.96 0.96 0.96 0.96 0.96
**Nursing Diagnosis/Outcome** Muscle Strength, Reduced Muscle Strength, Increased **Nursing Intervention** Assess Muscle Tone Assessing Nutrition Status (10030660) Promoting Exercise (10040834) Measure (or Check) Muscle Strength with a Dynamometer	0.96 0.96 1.00 0.96 0.96 0.93
**Nursing Diagnosis/Outcome** Impaired vision(10022748) Improved vision (10047353) **Nursing Intervention** Identifying Obstruction to Communication (10009683) Refer to Ophthalmologist Assessing Vision (10050138) Promoting Use of Glasses (10037643)	0.93 0.93 1.00 0.96 0.96 0.96
**Nursing Diagnosis/Outcome** Fear of Falling Fear of Falling, Reduced **Nursing Intervention** Advise on Fear of Falling Reassure Patient About Safety Facilitating Ability to Communicate Feelings (10026616) Assessing Fear of Falling	0.89 0.89 0.96 0.96 0.93 0.93
**SELF-CARE REQUIREMENTS RELATED TO HEALTH DEVIATIONS**	
**Nursing Diagnosis/Outcome** Self-medication Self-medication, Absent Self-medication, Reduced **Nursing Intervention** Advise on Risk Behavior Warn about the risks of self-medication Obtain Data on Self-Medication Monitor Self-Medication	0.96 0.96 0.96 1.00 1.00 0.96 0.93
**Nursing Diagnosis/Outcome** Functional Deficit Functional Deficit, Decreased **Nursing Intervention** Encourage Independence Assess Degree of Disability (or Limitation) Facilitating Activities of Daily Living (10051125) Reinforcing Capabilities (10026436) Assess Need for Corrective Device	0.96 0.96 1.00 0.96 0.96 0.96 0.89
**Nursing Diagnosis/Outcome** Impaired Walking (10001046) Able to Walk (10002833) **Nursing Intervention** Assisting with Walking (10038986) Assessing Ability to Walk (10038917) Recommend Use of Appropriate Footwear Monitor Gait (Walk) Investigate Dysmetria	0.96 0.96 0.96 0.96 0.96 0.93 0.89
**Nursing Diagnosis/Outcome** Chronic, Pain (10000546) No Pain (10029008) Reduced Pain (10027917) **Nursing Intervention** Evaluating Response to Pain Management (10034053) Implementing Comfort Care (10039705) Assessing Pain (10026119) Teaching About managing Pain (Control) (10019489) Administering Medication (10025444)	0.93 0.93 0.93 0.96 0.96 0.96 0.96 0.89
**Nursing Diagnosis/Outcome** Fall (10029405) No Fall (10034704) **Nursing Intervention** Guide the Family on Recording the Date and Time of the Fall Teaching Abou Fall Prevention (10040253) Listen to Older People Self-Report about the Circumstance of the Fall Assessing Knowledge of Fall Prevention (10039780) Post-Fall Evaluation (10037540)	0.89 0.89 1.00 1.00 0.96 0.93 0.89

## DISCUSSION

This study proposed the construction and validation of a terminological subset of the ICNP^®^ for the prevention of falls in elderly people in PHC. Although other terminological subsets have been developed for care for the elderly,^(8,9,19)^ in Brazil, this is the first to specifically address falls prevention in this target audience, demonstrating its relevance and originality.

The theoretical support adopted in the construction of this terminological subset was SCDT^(6)^, whose main concepts permeated and guided the stages of the study. This theory was also the basis for the development of other Brazilian subsets, for example, for elderly women with vulnerability related to HIV/AIDS^(10)^ and to care for people with leprosy^(20)^.

Although experiences of using SCDT in the construction of subsets in Brazil are identified, there is still a lack of international studies that use the theory in the construction of these instruments. An example of the use of SCDT was in the construction of the terminological subset intended for prenatal care, developed in China, in accordance with ICN recommendations^(21)^. Furthermore, it is worth highlighting that SCDT is not the only theory that contemplates the practice of self-care; another example is the Medium-Range Theory of Self-Care in Chronic Diseases, which was used as a theoretical framework for the subset of ICNP diagnoses^®^ for individuals with diabetes mellitus in Italy^(22)^.

In the present work, 182 ND/NO and 321 NI were constructed, with a predominance of concepts contained in ICNP^®^, demonstrating that its most current version addresses relevant concepts to describe the prevention of falls in older people. As for the non-constant concepts (58 ND/NO and 92 NI), these were constructed with the support of specialized terminology and ICNP^®^.

Considering that nursing care for elderly people susceptible to falls demands a comprehensive approach, it was found that SCDT can benefit from recognizing the imbalance between the capabilities to perform self-care and the therapeutic demands of self-care, identified through the self-care requirements proposed by Orem. It is understood that this moment is the starting point for nurses’ actions, as it will favor the tracking of risk factors for falls.

In this regard, based on the theoretical support adopted, the ND/NO were organized according to self-care requirements and the NI followed the classification of the corresponding ND/NO. There was a predominance of statements classified as health deviation requirements, followed by universal and development requirements.

The health diversion requirements included ND/NO relating to the experiences of people who are ill or injured, have disabilities, who have received a medical diagnosis, or are in the process of treatment. Statements such as “Self-medication”, “Confusion”, “Acute Pain”, “Impaired Walking”, “Paralysis” and “Tremor” were included. These, therefore, reflect developments in pathological conditions and disabilities, which can favor the occurrence of falls and deserve special attention from nurses to promote the fulfillment of therapeutic self-care demands.

As for the ND/NO of universal requirements, they refer to the integrity of human structural or functional aspects, including maintenance of water consumption (e.g.: “Dehydration”, “Hydration, Adequate”), air (e.g.: “Dyspnea”, “Dyspnea, Absent”) and food (e.g.: “Obesity”, “Weight, within Normal Limits”); balance between activity and rest (e.g. “Physical Exercise Behavior, Impaired”, “Impaired Sleep”) and provision of care related to eliminations (e.g. “Diarrhea”, “Urinary Incontinence”). Furthermore, they address the prevention of risks to life and well-being (e.g.: “Self-Care Deficit”, “Impaired Health Maintenance”, “Risk for Fall”); and the promotion of human functioning and development within social groups (e.g. “Positive Family Support”, “Lack of Family Support”).

It is observed that this set of requirements represents needs that are common to human beings, regardless of the life cycle, but which can have a more intense impact on the elderly, depending on their capabilities/limitations for self-care. In this sense, it is essential that nurses incorporate the systematic assessment of these aspects in nursing consultations with a view to identifying self-care deficits and, as a result, developing the care plan and implementing nursing interventions aimed at minimizing or resolving them. them, from the perspective of preventing fall episodes.

Regarding development requirements, it was considered that they promote life and maturation processes, associated with events in the aging process. These include, for example, the ND/NO “Impaired Attention”, “Impaired Psychomotor Activity”, “Impaired Cognition”, “Impaired Balance”, “Muscle Strength, Reduced” and “Impaired Vision”. These changes are characteristic of human aging, requiring older people’s ability to adapt to maintenance of independence and safety. Therefore, it is up to the nurse to assess how much these changes interfere with the ability of the older person/family to carry out self-care actions and mobilize skills to overcome the identified deficits, implementing care in the context of nursing systems and with the health team.

The classification of ND/NO according to self-care requirements was a challenge due to the complexity of the theory and its high level of abstraction, characteristic of major nursing theories^(23)^. Similar proposals were observed in other studies on the national^(10,20)^ and international^(24)^ scene; however, differences were observed in the classifications of statements, indicating the need for authors to record their understanding of the self-care requirements that guided the development of their research. Furthermore, the difficulties in organizing statements according to self-care requirements explain the changes in the first classification mentioned (that is, in the classification that preceded content validation), carried out according to experts’ suggestions.

After the construction of the ND/NO, the operational definitions for the ND/NO were developed/adjusted. It should be noted that these definitions are essential for determining ND/NO, as they describe their meanings and how they will be measured, besides increasing the reliability and validity of clinical data related to ND/NO and favoring the development and replication of researches^(25)^.

With regard to NI, the construction of the statements resulted from reflection on each ND/NO of the different self-care requirements and on the role of the nurse in PHC and in preventing falls in older people, with support from the literature. This process resulted in a broad list of NIs designed, for example, to assess the risk of falls, carry out home visits, health education to prevent falls, promote physical exercise and collaborate with members of the multidisciplinary team^(4)^.

It should be noted that the professional must select the NI most appropriate to the singularities of the older person and implement them considering the nursing systems (educational support, partially compensatory, and fully compensatory). When constructing the subset, it was decided not to designate in which system the NI will be used, as it is understood that the same intervention can be implemented in different nursing care settings, and it is up to the nurse to develop this reasoning.

To reflect on the application of ND/NO/NI, a study developed in Maringá-PR was used, in which researchers assessed the risk of falls in older women in the PHC setting, determined the main NDs related to falls and developed intervention plans, based on ICNP^®^ and in the International Classification of Nursing Practices in Public Health (ICNPHH^®^). The following NDs were identified: weight loss, urinary incontinence, inadequate physical activity, impaired walking, decreased manual dexterity, weakness, poor memory, risk of domestic accidents – older people, lack of knowledge about fall prevention and risk of falling. For each ND, a list of possible NI was listed^(26)^.

Given the statements proposed in the research^(26)^, we can see the coherence with the NO/NO/NI that make up this terminological subset. Some statements were expressed with different terms, for example, the ND “impaired walking”, corresponding to “impaired gait”; and the NI “tracking the risk of falls”, similar to “obtaining data on the risk of falls”, aspects that reinforce the relevance of the subset to standardize the language of professionals in assistance and research development.

Regarding the characteristics of specialist nurses, the area of professional activity was highlighted, with a predominance of teaching, research and extension, revealing a profile of nurses who work in the academic area. And the representation of professionals from the five regions of Brazil, with emphasis on the Northeast, Central-West and South, allows us to infer that the statements have the potential to describe nursing practice in preventing falls in older people in PHC in different realities in the country.

Regarding the largest number of participants from the Northeast, it is noteworthy that this region is the one that most concentrates research on the development of ICNP terminological subsets^®^ in the country. An integrative review published in 2019 showed that of the total of 35 subsets built over a ten-year period, the majority (57.14%) were developed in universities located in the Northeast. One of the factors that explains this characteristic is the existence of the Brazilian ICNP^®^ Research and Development Center, linked to the Postgraduate Program in Nursing at the Federal University of Paraíba^(27)^.

The results of validating the statements showed that all ND/NO/NI achieved a CVI score sufficient for validation (CVI ≥ 0.80); this finding differs from the experience of other similar studies whose data collection was carried out using electronic forms^(28,29)^.

In the present study, it is assumed that the experts’ selection criteria may have influenced the evaluation of the statements. It was observed that nurses with experience in research related to ICNP^®^ and/or gerontology, even if they do not have expertise in the approach to falls in the PHC context, they achieved the minimum score required to participate in the study.

In relation to the organization of the statements to compose the terminological subset, it consisted of the systematization of the constructed knowledge, aiming at making the information tangible for the nurse who will benefit from its content. It was decided that the NDs with corresponding NO would be presented, along with an NI relationship for achieving the result; in addition, the organization remained in accordance with self-care requirements. These choices were designed so that the nurse can envision the application of the subset in light of SCDT; in clinical practice, the determination of concepts as ND or NO, as well as the selection of NI, must be carried out according to the characteristics/needs of the older person and family.

Regarding the contributions of the present study, the stimulus for the application of ICNP^®^ by nurses is highlighted, favoring clinical judgment in assisting individuals, since its structure can be improved with indicators that reflect the reality of practice^(30)^. Furthermore, it allowed the construction of an unprecedented information instrument with the potential to direct nursing care to prevent falls in older people; improve the quality of records by adopting standardized language; benefit the teaching and learning process and the development of nursing research.

A limitation is the failure to perform cross-mapping of statements from the ICNP^®^ terminological subset with SNOMED - *Clinical Terms* (SNOMED CT) statements, especially due to the lack of translation of this controlled vocabulary into Portuguese. Although SNOMED CT is not yet a reality in health institutions in Brazil, further studies are required to better assimilate its interoperability with other standardized language systems, including ICNP^®^.

## CONCLUSION

The methodological steps presented allowed the construction and validation of the “ICNP^®^ Terminological Subset for preventing falls in the elderly in the context of primary health care”, in light of SCDT, meeting the proposed objective. In the first stage, 182 ND/NO and 321 IE were built. In the second stage, the constructed statements had their content validated by specialist nurses. The final organization of the statements showed 92 ND, 90 NO, and 321 NI, with a predominance of statements related to health deviation requirements. The proposition of this instrument cooperates with the ICN recommendation for the construction of terminological subsets that contribute to the description of professional practice through standardized languages and stands out for being the first subset for the prevention of falls in older people in PHC, representing a starting point for evaluations and planning of future studies.
